# Prediction of prognosis in elderly patients with sepsis based on machine learning (random survival forest)

**DOI:** 10.1186/s12873-022-00582-z

**Published:** 2022-02-11

**Authors:** Luming Zhang, Tao Huang, Fengshuo Xu, Shaojin Li, Shuai Zheng, Jun Lyu, Haiyan Yin

**Affiliations:** 1 Intensive Care Unit, The First Affiliated Hospital of Jinan University, Guangzhou, 510630 People’s Republic of China; 2 Department of Clinical Research, The First Affiliated Hospital of Jinan University, Guangzhou, Guangdong Province China; 3 School of Public Health, Xi’an Jiaotong University Health Science Center, Xi’an, Shaanxi Province China; 4 Department of Orthopaedics, The First Affiliated Hospital of Jinan University, Guangzhou, Guangdong Province China; 5 School of Public Health, Shannxi University of Chinese Medicine, Xianyang, Shaanxi Province China

**Keywords:** Machine learning, Random survival forest, Elderly, Sepsis, Prognosis

## Abstract

**Background:**

Elderly patients with sepsis have many comorbidities, and the clinical reaction is not obvious. Thus, clinical treatment is difficult. We planned to use the laboratory test results and comorbidities of elderly patients with sepsis from a large-scale public database Medical Information Mart for Intensive Care (MIMIC) IV to build a random survival forest (RSF) model and to evaluate the model’s predictive value for these patients.

**Methods:**

Clinical information of elderly patients with sepsis in MIMIC IV database was collected retrospectively. Machine learning (RSF) was used to select the top 30 variables in the training cohort to build the final RSF model. The model was compared with the traditional scoring systems SOFA, SAPSII, and APSIII. The performance of the model was evaluated by C index and calibration curve.

**Results:**

A total of 6,503 patients were enrolled in the study. The top 30 important variables screened by RSF were used to construct the final RSF model. The new model provided a better C-index (0.731 in the validation cohort). The calibration curve described the agreement between the predicted probability of RSF model and the observed 30-day survival.

**Conclusions:**

We constructed a prognostic model to predict a 30-day mortality risk in elderly patients with sepsis based on machine learning (RSF algorithm), and it proved superior to the traditional scoring systems. The risk factors affecting the patients were also ranked. In addition to the common risk factors of vasopressors, ventilator use, and urine output. Newly added factors such as RDW, type of ICU unit, malignant cancer, and metastatic solid tumor also significantly influence prognosis.

**Supplementary Information:**

The online version contains supplementary material available at 10.1186/s12873-022-00582-z.

## Background

Despite the growing awareness of sepsis, advanced diagnostic methods, broad-spectrum antibiotics, and intensive care, sepsis remains a major public health problem worldwide [1]. Most epidemiological studies on sepsis come from developed countries. It is estimated that worldwide, about 30 million patients are affected by sepsis each year, of which about 5 million patients die [2], accounting for about 20% of global deaths [3]. With the aggravation of the aging society, the incidence of sepsis in the elderly is gradually increasing; sepsis is among the diseases that lead to the highest mortality among elderly patients [4]. Elderly patients have low immunity [5], reduced organ reserve function, comorbidities such as diabetes and coronary heart disease are more common than younger patients [6], and atypical clinical symptoms after infection; thus, it is easy to miss diagnosis or for a misdiagnosis to occur. Sepsis occurs and quickly progresses to multiple organ failure [7]. Thus, the clinical mortality rate is high. In addition, changes in the pharmacokinetics of elderly patients have also made the treatment of sepsis difficult [8]. Furthermore, a prospective cohort study haven illustrated that older sepsis survivors bear a higher burden of persistent disability and 12-month mortality compared with younger patients [9]. Other researches also very clearly demonstrated, elderly patients with sepsis are more likely to have long-term cognitive impairment and dysfunction [10, 11].

The development of medical information technology and the popularization of electronic medical record system provide the basis for the clinical application and evaluation of a prognostic model. Random survival forest (RSF) is a machine learning method based on decision trees. The algorithm uses internal data cross-validation to ensure high prediction accuracy without over-fitting, which is suitable for survival analysis of many diseases [12, 13]. The RSF model need not assume that variable for the influence of the risk function is linear, in addition to this, it can also rank the importance of variables, so as to screen variables with greater importance and reduce the dimension of variables [14, 15], which is beneficial to the application of the model in clinical practice [16, 17]. Maryam et al. have illustrated this point clearly, their research showed that the machine learning prediction model can well predict the major adverse cardiac and cerebrovascular events during long-term follow-up after percutaneous coronary intervention [18]. Sequential Organ Failure Assessment (SOFA), Simplified acute physiological score II (SAPSII), and Acute physiology score III (APSIII)  [19, 20] contain the evaluation of multiple laboratory indicators, which are often used to predict the prognosis of diseases, but they still have certain limitations. Current studies tend to add some new markers on the basis of the abovementioned scoring system [21, 22], or reconstruct the scoring system [23], to improve their performance in predicting disease prognosis.

Researches have shown that early identification and assessment of sepsis is key to improving survival in older patients with sepsis [24, 25]. At present, no study has used the RSF model to predict the prognosis of elderly patients with sepsis. We planned to use the laboratory test results and comorbidities of elderly patients with sepsis from the large-scale public database MIMIC IV to build the RSF model and evaluate its predictive value for elderly patients with sepsis.

## Methods

### Data source and study population

The MIMIC-IV v0.4 database is a large public database that contains hospitalization information for patients at Beth Israel Deaconess Medical Center between 2008 and 2019, which was approved by the Massachusetts Institute of Technology (Cambridge, MA) and Beth Israel Deaconess Medical Center (Boston, MA). Because the present study was an analysis of the third party anonymized publicly available database with pre-existing institutional review board (IRB) approval, our institution’s IRB approval was exempted. This database provides a strong information base for clinical studies. In the database, the true identity information about the patient is hidden. Thus, obtaining the patient’s informed consent was not needed. The author completed the relevant course training and obtained the certificate to access the database. All data are from Physionet official website (https://mimic.physionet.org/).

A total of 11,897 patients were diagnosed with sepsis in the database, including 6,567 patients aged 65 years old or older. Exclusion criteria were as follows: patients who died within 24 h of entering intensive care unit (ICU). Finally, a total of 6,503 patients were selected for the study.

### Data extraction

Using Structured Query Language to extract data, the extracted variables included the general information of patients, as follows: ethnicity, sex, age, weight, ventilator use, vasopressor use, continuous renal replacement therapy (CRRT) use, and first care unit (unit). The severity of the disease was assessed using SOFA, SAPS II, and APS III. Charlson comorbidity index was used, and the comorbidities included the following: myocardial infarction, congestive heart failure, peripheral vascular disease, cerebrovascular disease, dementia, chronic pulmonary disease, rheumatic disease, peptic ulcer disease, mild liver disease, diabetes uncomplicated, diabetes complicated, paraplegia, renal disease, malignant cancer, severe liver disease, metastatic solid tumor, and AIDS. Results of the first laboratory examination after admission to the ICU included data on the following: white blood cells (WBC), red blood cells (RBC), hemoglobin, hematocrit, red cell distribution width (RDW), mean corpuscular hemoglobin (MCH), mean corpuscular hemoglobin concentration (MCHC), mean corpuscular volume (MCV), platelet count (PLT), prothrombin time (PT), partial thromboplastin time (PTT), INR PT, lactate, calculated total CO_2_, PaCO_2_, pH, PaO_2_, alanine aminotransferase (ALT), aspartate aminotransferase (AST), albumin, alkaline phosphatase(AP), bilirubin total, urea nitrogen, creatinine, glucose, anion gap (AG), base excess, calcium total, chloride, magnesium, bicarbonate, phosphate, potassium, sodium, specific gravity, urine output. Vital signs included data on the following: mean heartrate, mean systolic blood pressure, mean diastolic blood pressure, mean blood pressure, mean respiratory rate, mean temperature, and mean SpO_2_.

### Statistical analysis

In this study, indicators with a missing degree greater than 20% were not included, and the remaining missing data were filled in by multiple imputation. In this study, the final complete data was generated from 10 imputed datasets obtained by the "mice" package of the R software [26].

The elderly patients with sepsis were randomly assigned to the training cohort (80%) or validation cohort (20%). The training cohort was used to construct the RSF model and perform internal validation. The validation cohort was used to verify the performance of the model. Categorical variables were described by frequency and percentage values, and differences between cohorts were determined by the chi-square test or Fisher's exact test. In some statistical guides, it is shown that for descriptive statistics, the median and quartiles are preferred over means and standard deviation values [27]. Therefore, in this study, the median and quartiles are used to describe continuous variables.

RSF is an ensemble method [28], which firstly uses the Bootstrap's sampling method to randomly select N samples from the training cohort to generate N survival trees, and then at each node of the tree, randomly select a subset of the covariates as candidate variables for splitting. Therefore, each tree is composed of categorized or split node variables, where tree nodes are split according to the maximum survival difference between child nodes, which can be calculated by four methods, namely log-rank, conservation of events, log-rank score, and random [15]. The method used in this study is the log-rank. For each bootstrap sample, about 37% of the samples in the training cohort were not extracted on average, and these samples were called out-of-bag (OOB) samples. The OOB error rate of the OOB sample was calculated. The OOB error rate and the predictive error rate of the validation set were used to evaluate the model’s performance. The lower the error rate was, the better the model performance was. In this study, the optimal parameter combination of the model was determined by calculating the error rate of the bag in the training cohort under various parameter combination conditions through grid search [29]. The parameter combination that made the total error rate of the RSF the lowest was determined. RSF model was built according to the optimal parameters, and variables were screened according to variable importance (VIMP)^14^. The importance score is an evaluation index used to measure the predictive ability of predictive variables to outcome variables. The greater the VIMP value was, the stronger the predictive ability was. VIMP was positive, indicating that the variable had a predictive effect. A VIMP of 0 or a negative value indicated that the variable was not a meaningful predictor. Ranking was performed according to the score of order of importance from the most important to the least important. The top 30 variables of importance were selected, and the RSF was built again. C index and calibration curves were used to evaluate the performance of the model.

In this study, data analysis was performed using R 4.0.3 software and Python 3.7; the packages used include randomForestSRC, survival, survivalROC, matplotlib, and scikit-learn.

## Results

Of 6,503 elder sepsis patients, 5,202 were in the training cohort, and 1,301 were in the validation cohort. The median age of the training cohort was 77.00 (70.00, 83.00), and the median age of the validation cohort was 76.00 (70.00, 83.00). Male patients accounted for 49.9% in the training cohort and 49.4% in the validation cohort. The median weight of patients in the training cohort was 75.00 (63.30, 89.88), and that in the validation cohort was 73.30 (61.90, 88.60). Among the comorbidities, renal disease accounted for the largest proportion, which was 30.5% in the training cohort and 30.0 in the validation cohort. Other baseline characteristics are shown in Table 1.

**Table 1:** Baseline characteristics of the patients

	Training Cohort	Validation Cohort
n	5202	1301
Age,year	77.00 (70.00, 83.00)	76.00 (70.00, 83.00)
Sex (%)		
Male	2596 (49.9)	643 (49.4)
Female	2606 (50.1)	658 (50.6)
Weight,kg	75.00 (63.30, 89.88)	73.30 (61.90, 88.60)
First care unit (%)		
MICU/SICU	2699 (51.9)	668 (51.3)
TSICU	1210 (23.3)	306 (23.5)
CCU	1104 (21.2)	274 (21.1)
Other	189 ( 3.6)	53 ( 4.1)
Ethnicity (%)		
White	3693 (71.0)	902 (69.3)
Black	492 ( 9.5)	121 ( 9.3)
Other	1017 (19.6)	278 (21.4)
Ventilator (%)		
No	1753 (33.7)	441 (33.9)
Yes	3449 (66.3)	860 (66.1)
Vasopressor (%)		
No	2917 (56.1)	720 (55.3)
Yes	2285 (43.9)	581 (44.7)
CRRT(%)		
No	4866 (93.5)	1228 (94.4)
Yes	336 ( 6.5)	73 ( 5.6)
Comorbidity		
Myocardial infarct (%)		
No	4204 (80.8)	1034 (79.5)
Yes	998 (19.2)	267 (20.5)
Congestive heart failure (%)		
No	3184 (61.2)	796 (61.2)
Yes	2018 (38.8)	505 (38.8)
Peripheral vascular disease (%)		
No	4449 (85.5)	1123 (86.3)
Yes	753 (14.5)	178 (13.7)
Cerebrovascular disease (%)		
No	4334 (83.3)	1108 (85.2)
Yes	868 (16.7)	193 (14.8)
Dementia (%)		
No	4748 (91.3)	1184 (91.0)
Yes	454 ( 8.7)	117 ( 9.0)
Chronic pulmonary disease (%)		
No	3751 (72.1)	912 (70.1)
Yes	1451 (27.9)	389 (29.9)
Rheumatic disease (%)		
No	4983 (95.8)	1253 (96.3)
Yes	219 ( 4.2)	48 ( 3.7)
Peptic ulcer disease (%)		
No	5033 (96.8)	1255 (96.5)
Yes	169 ( 3.2)	46 ( 3.5)
Mild liver disease (%)		
No	4718 (90.7)	1179 (90.6)
Yes	484 ( 9.3)	122 ( 9.4)
Diabetes uncomplicated (%)		
No	3818 (73.4)	996 (76.6)
Yes	1384 (26.6)	305 (23.4)
Diabetes complicated (%)		
No	4600 (88.4)	1163 (89.4)
Yes	602 (11.6)	138 (10.6)
Paraplegia (%)		
No	4918 (94.5)	1237 (95.1)
Yes	284 ( 5.5)	64 ( 4.9)
Renal disease (%)		
No	3613 (69.5)	911 (70.0)
Yes	1589 (30.5)	390 (30.0)
Malignant cancer (%)		
No	4468 (85.9)	1116 (85.8)
Yes	734 (14.1)	185 (14.2)
Severe liver disease (%)		
No	5005 (96.2)	1243 (95.5)
Yes	197 ( 3.8)	58 ( 4.5)
Metastatic solid tumor (%)		
No	4895 (94.1)	1233 (94.8)
Yes	307 ( 5.9)	68 ( 5.2)
AIDS (%)		
No	5196 (99.9)	1297 (99.7)
Yes	6 ( 0.1)	4 ( 0.3)
Laboratory tests		
WBC (k/uL)	11.80 (8.20, 16.70)	11.80 (8.00, 16.80)
RBC (m/uL)	3.36 (2.93, 3.85)	3.38 (2.93, 3.88)
Hemoglobin (g/dL)	10.00 (8.70, 11.40)	10.00 (8.70, 11.50)
Hematocrit (%)	30.90 (27.10, 35.10)	31.00 (27.00, 35.20)
RDW (%)	15.20 (14.10, 16.90)	15.30 (14.10, 16.80)
MCH (IU/mL)	30.10 (28.40, 31.50)	30.10 (28.60, 31.40)
MCHC (%)	32.50 (31.40, 33.60)	32.50 (31.30, 33.50)
MCV (fL)	92.00 (88.00, 97.00)	92.00 (88.00, 97.00)
PLT(k/uL)	191.00 (133.00, 266.00)	193.00 (136.00, 269.00)
INR PT (s)	1.30 (1.20, 1.70)	1.30 (1.20, 1.70)
PT (s)	14.80 (13.00, 18.50)	14.80 (12.90, 18.30)
PTT (s)	32.10 (27.90, 40.20)	32.20 (28.20, 40.20)
Lactate (mmol/L)	1.70 (1.20, 2.60)	1.70 (1.20, 2.60)
Calculated Total CO2	24.00 (20.00, 28.00)	24.00 (21.00, 28.00)
PaCO2 (mmHg)	40.00 (34.00, 47.00)	40.00 (34.00, 47.00)
pH	7.37 (7.30, 7.43)	7.38 (7.31, 7.43)
PaO2 (mmHg)	86.00 (50.00, 155.00)	86.00 (50.00, 161.00)
ALT (IU/L)	25.00 (15.00, 52.00)	24.00 (15.00, 50.00)
AST (IU/L)	34.50 (22.00, 73.00)	34.00 (23.00, 68.00)
Albumin (mg/dL)	2.90 (2.40, 3.30)	2.90 (2.50, 3.20)
AP (IU/L)	90.00 (64.00, 132.75)	90.00 (64.00, 139.00)
Bilirubin Total (mg/dL)	0.60 (0.40, 1.20)	0.60 (0.40, 1.20)
Urea Nitrogen (mg/dL)	28.00 (18.00, 45.00)	27.00 (18.00, 44.00)
Creatinine (g/dL)	1.20 (0.80, 1.90)	1.20 (0.80, 1.90)
Glucose (mg/dL)	132.00 (105.00, 172.00)	130.00 (104.00, 172.00)
AG (mEq/L)	15.00 (12.00, 18.00)	15.00 (12.00, 18.00)
Base Excess(mEq/L)	-1.00 (-5.00, 1.00)	-1.00 (-5.00, 1.00)
Calcium Total (EU/dL)	8.20 (7.70, 8.70)	8.20 (7.70, 8.70)
Chloride (mEq/L)	104.00 (100.00, 109.00)	104.00 (100.00, 109.00)
Magnesium (mg/dL)	1.90 (1.70, 2.20)	1.90 (1.70, 2.20)
Bicarbonate (mEq/L)	22.00 (19.00, 26.00)	22.00 (19.00, 26.00)
Phosphate (mg/dL)	3.60 (2.90, 4.40)	3.60 (2.90, 4.40)
Potassium (mEq/L)	4.10 (3.70, 4.60)	4.10 (3.70, 4.60)
Sodium (mEq/L)	139.00 (136.00, 142.00)	139.00 (136.00, 142.00)
Specific Gravity	1.02 (1.01, 1.02)	1.02 (1.01, 1.02)
urineoutput (ml)	1180.00 (685.00, 1860.00)	1170.00 (700.00, 1875.00)
Vital Signs		
Mean heartrate (min-1)	84.60 (74.19, 96.95)	85.56 (75.64, 97.23)
Mean systolic blood pressure (mmHg)	112.15 (104.08, 123.71)	112.04 (103.65, 123.23)
Mean diastolic blood pressure (mmHg)	57.75 (51.91, 64.19)	57.33 (51.27, 64.06)
Mean blood pressure (mmHg)	72.78 (67.32, 79.44)	72.69 (67.04, 79.24)
Mean respiratory rate (min-1)	19.85 (17.38, 22.68)	19.83 (17.47, 22.58)
Mean temperature (℃)	36.80 (36.54, 37.13)	36.80 (36.53, 37.10)
Mean SpO2 (%)	97.15 (95.75, 98.44)	97.21 (95.79, 98.48)

### Modeling process

**Fig.1:**
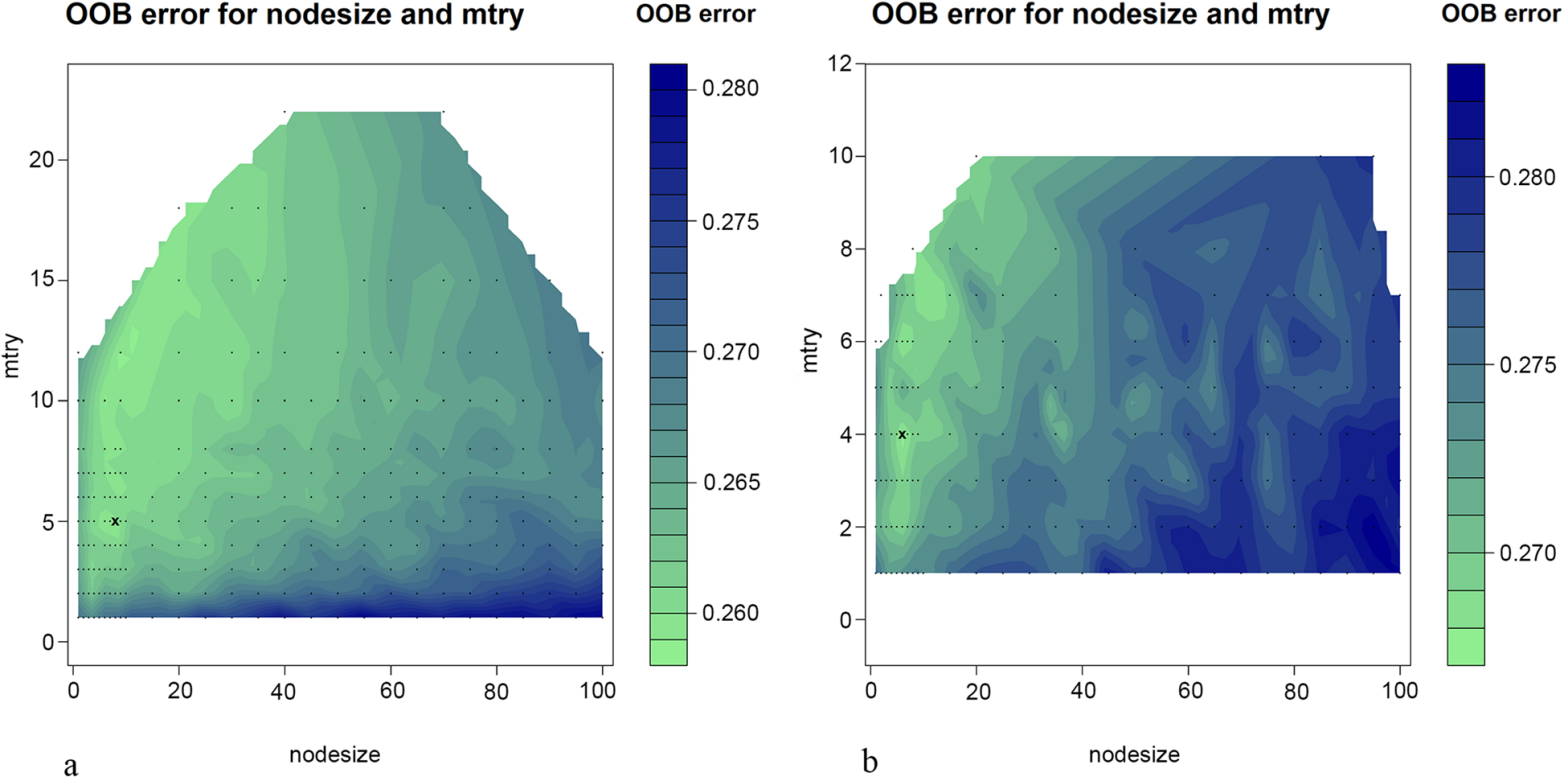
Tuning parameters of RSF model

**Fig. 2:**
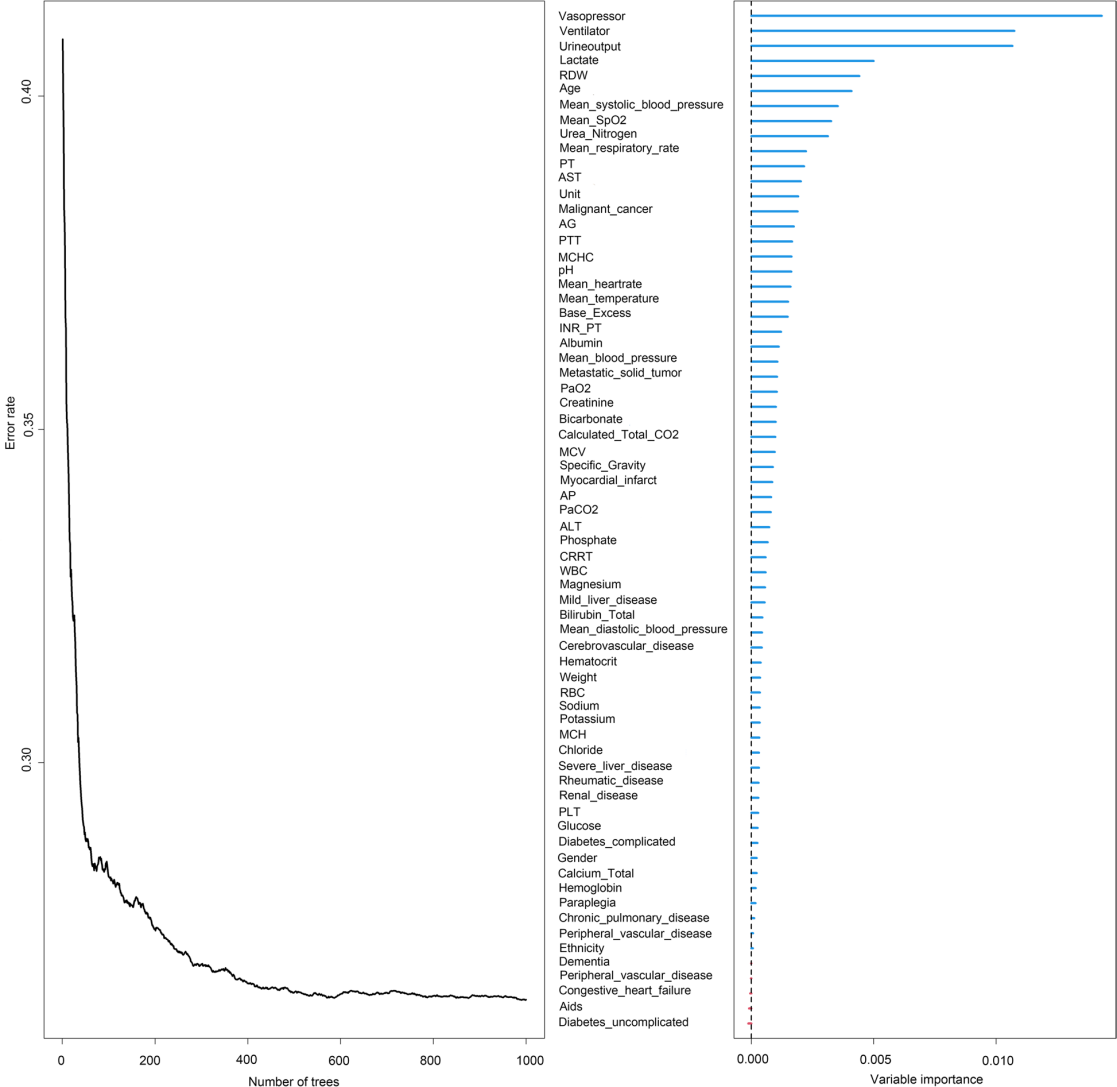
Variable importance and error rate curve of RSF

We calculated the OOB error rate in the training cohort under various mtry and nodesize combinations by grid search. As shown in Fig. 1a, under the parameter combination condition of mtry = 8 and nodesize = 5, the OOB error rate of the model in the training cohort reached the lowest rate (26.35%), and the OOB error rate of the model tended to be stable at 1000 survival trees. The top 30 variables in the importance diagram of variables (Fig. 2, Supplementary material) were selected to build a random forest model. The optimal mtry = 4 and nodesize = 8 were determined again in the same way (Fig. 1b), and the OOB was 27.30%, and these values were used to build a random forest model.

### Modeling validation

**Fig. 3:**
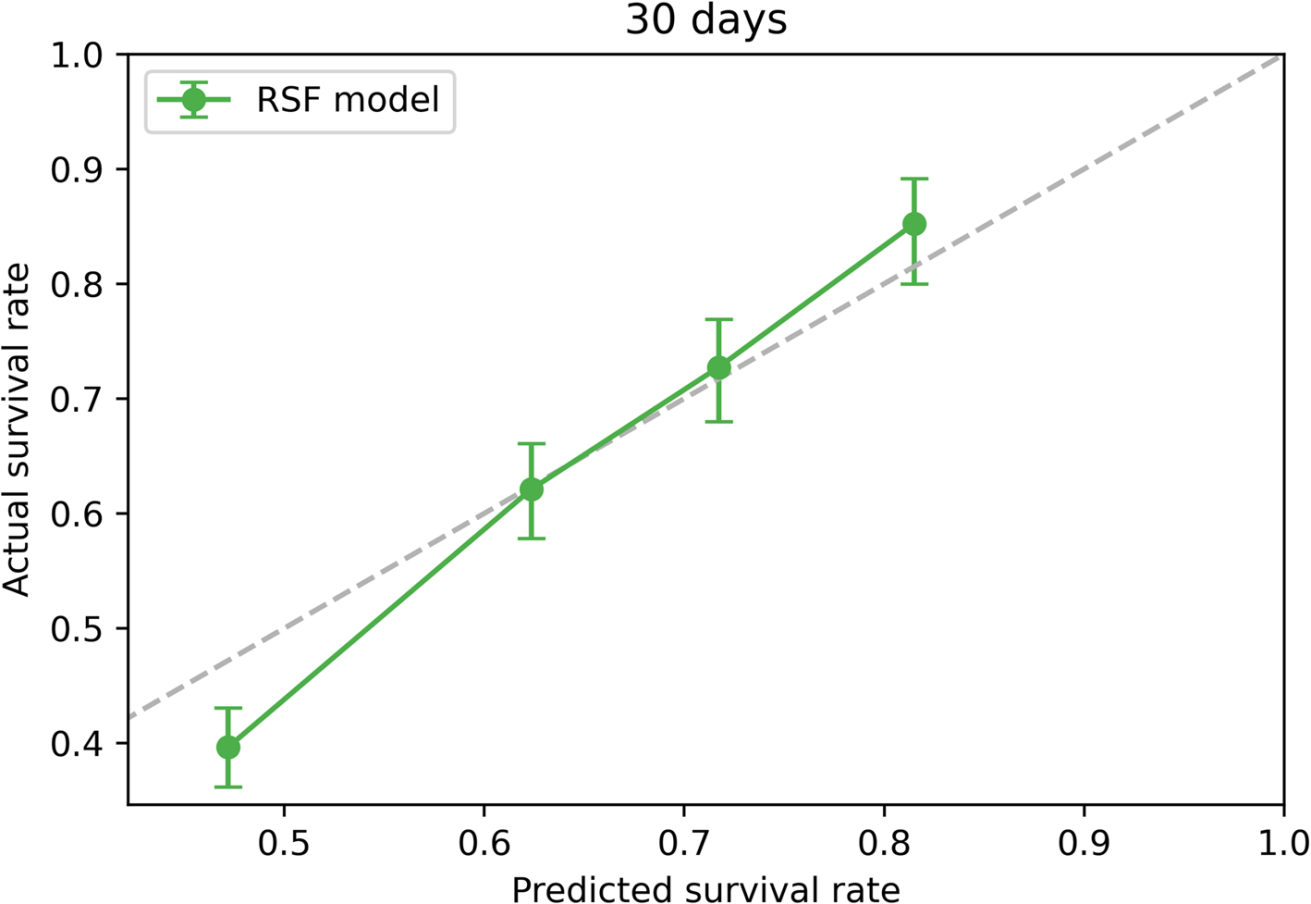
Calibration curves for the validation cohort

The C indexes of the four models (SOFA, SAPSII, APSIII, and RSF) in the validation cohort were as follows: 0.551, 0.654, 0.669, and 0.731, respectively. The calibration curve described the calibration of the RSF model, that is, the agreement between the predicted probability and the observed 30-day survival (Fig. 3).

## Discussion

In this present study, we established a prognostic prediction model for predicting 30-day mortality risk in elderly patients with sepsis based on the machine learning (RSF), which can provide a basis for clinical decision-making. Our model is unique, it ranked clinically common laboratory examinations and comorbidities according to the variable importance through RSF, and selected the top 30 variables to build the final RSF model, which is not done in traditional scoring systems. Moreover, we used C index to compare the RSF model with the traditional SOFA, SAPSII, and APSIII scoring system, showing RSF exhibits better predictive performance. The calibration curve further confirmed that the newly constructed RFS model could be used to predict 30-day mortality in elderly patients with sepsis.

Among the variables related to the prediction of sepsis in elderly patients, the top variables are the use of vasopressor, the use of ventilator, the patient’s urine output during the first 24 h, lactate level, and mean systolic blood pressure 24 h after entering the ICU. These are important indicators that can be used to evaluate whether circulatory disorders, respiratory disorders, and other organ dysfunctions occur in elderly patients with sepsis [30, 31]. In addition to lactate, these abovementioned top indicators are also found in SOFA, SAPSII, and APSIII scoring systems, indicating their importance for disease prediction [32]. In recent years, the number of studies about the prognosis of lactate in sepsis has been increasing, because lactate can reflect the degree of hypoxia in patients. For example, one study showed that early detection of lactate was associated with 28-day mortality from sepsis [33].

RDW and the type of ICU unit are some of the new indicators added to the RFS model, which are not included in the traditional scoring system. In recent years, RDW has been of great value as a marker of poor prognosis for diseases of the nervous system, cardiovascular system, and other systemic systems [34–36]. The increased value of RDW can indirectly reflect the imbalance of RBC homeostasis, which may be due to the impaired RBC formation ability and abnormal RBC survival caused by the body’s abnormal metabolism [37]. The abovementioned changes in RBC may be due to the large number of inflammatory factors produced in the process of severe metabolic disorder and oxidative stress reaction in sepsis patients.

The patient’s ICU unit reflects the difference in the etiology of sepsis, the more that is known about this the more specific therapies can be, so this also occupies an important part [38].Sepsis can arise from different causes, such as traumatic infection, postoperative infection, and severe pneumonia, which have different effects on the prognosis of patients [39]. These should receive recognition in clinical practice. In addition, malignant cancer and metastatic solid tumor are also new variables. The absolute value of neutrophils in malignant tumors or solid tumors is reduced by intensive cytotoxic chemotherapy, thereby reducing the survival rate of patients [40]. Moreover, the immune system dysfunction that tumors share with sepsis is also associated with lower survival rates in older patients with sepsis [41].

In short, we use RSF to overcome the weaknesses of traditional survival analysis methods to build a model with high predictive performance. With the advent of the medical big data era, machine learning models will be increasingly used in clinical practice to help improve the prognosis of patients [42].

### Strengths and limitations of the study

The advantage of this study is that it adopts machine learning method to construct an RSF model which is superior to traditional SOFA, SAPSII, and APSIII scoring system. At the same time, the importance of variables was ranked, so that clinicians can more intuitively understand the indicators that have a greater impact on the outcome. This study also has limitations, first of all, it is a single-center study and lacks external verification. Moreover, when machine learning is applied in clinical practice, the 30-day survival probability of elderly patients with sepsis can be predicted by creating web pages and inputting indicators in the model. One of our limitations is that a complete web page is not generated, which will be improved in future research.

## Conclusions

We constructed a prognostic model for predicting 30-day mortality risk in elderly patients with sepsis based on the machine learning (RSF algorithm), and it proved superior to the traditional scoring system. The risk factors affecting the patients were also ranked. In addition to the common risk factors of vasopressors, ventilator use, and urine output. Newly added factors such as RDW, type of ICU unit, malignant cancer, and metastatic solid tumor also significantly influence prognosis.

## Supplementary Information

**Additional file 1.**

## Data Availability

The data were available on the MIMIC-IV website at https://mimic.physionet.org/, https://doi.org/10.13026/a3wn-hq05.

## Abbreviations

MIMIC IV: Medical Information Mart for Intensive Care IV
RSF: Random survival forest; ICU: intensive care unit
SOFA: Sequential Organ Failure Assessment
SAPSII: Simplified acute physiological score II
APSIII: Acute physiology score III
IRB: Institutional review board
CRRT: Continuous renal replacement therapy
unit: First care unit
WBC: White blood cells
RBC: Red blood cells
RDW: Red cell distribution width
MCH: Mean corpuscular hemoglobin
MCHC: Mean corpuscular hemoglobin concentration
MCV: Mean corpuscular volume
PLT: Platelet count
PT: Prothrombin time
PTT: Partial thromboplastin time
ALT: Alanine aminotransferase
AST: Aspartate aminotransferase
AP: Alkaline phosphatase
AG: Anion gap
OOB: Out-of-bag
VIMP: Variable importance
